# Gallbladder bile supersaturated with cholesterol in gallstone patients preferentially develops from shortage of bile acids

**DOI:** 10.1194/jlr.S091199

**Published:** 2019-01-04

**Authors:** Mats Rudling, Amit Laskar, Sara Straniero

**Affiliations:** Metabolism Unit, Endocrinology, Metabolism and Diabetes, and Integrated CardioMetabolic Center (ICMC), Department of Medicine, Karolinska Institutet at Karolinska University Hospital, Huddinge, S-141 86 Stockholm, Sweden

**Keywords:** bile acid metabolism, bile physical chemistry/gallstone formation, cholesterol metabolism, gallstone formation, obesity

## Abstract

Gallstone (GS) formation requires that bile is supersaturated with cholesterol, which is estimated by a cholesterol saturation index (CSI) calculated from gallbladder (GB) total lipids and the mol% (mole percent) of bile acids (BAs), cholesterol, and phospholipids (PLs). Whereas CSI indicates GS risk, we hypothesized that additional comparisons of GB lipid mol% data are inappropriate to identify why CSI is increased in GS disease. We anticipated that GB lipid mmol/l (millimole per liter) levels should instead identify that, and therefore retrieved GB mmol/l data for BAs, cholesterol, and PLs from a study on 145 GS and 87 GS-free patients and compared them with the corresponding mol% data. BA and PL mmol/l levels were 33% and 31% lower in GS patients, while cholesterol was unaltered. CSI was higher in GS patients and correlated inversely with GB levels of BAs and PLs, but not with cholesterol. A literature search confirmed, in 13 studies from 11 countries, that GB BA levels and, to a certain extent, PLs are strongly reduced in GS patients, while cholesterol levels are not elevated. Our findings show that a shortage of BAs is a major reason why GB bile is supersaturated with cholesterol in GS patients. These results are sustainable because they are also valid from a global perspective.

Cholesterol gallstone disease (GSD) is a common multifactorial gastrointestinal condition. A prerequisite for gallstone (GS) development is that gallbladder (GB) bile is supersaturated with cholesterol (1–3). A common way to monitor this is from a cholesterol saturation index (CSI), calculated from the mol% (mole percent) values of the three major lipids in GB bile: bile acids (BAs), cholesterol, and phospholipids (PLs) together with the total lipids (4). CSI is an established indicator of GSD risk. However, a high CSI value does not indicate whether increased cholesterol levels, reduced BA levels, and/or reduced PL levels in GB bile are causing it, a crucial question for identification of the major most common cause for supersaturated bile. Nevertheless, a frequent view is that increased CSI in GB bile is primarily due to hepatic hypersecretion of cholesterol into bile (5–10). In studies on lipid levels in GB bile, authors often compare mol% data of GB lipids from patients with and without GSs. From such comparisons, authors frequently conclude that the mol% of cholesterol is increased in GB bile from patients with GSD, a finding often considered to support that hypersecretion of cholesterol is the primary and major cause for why GB bile from patients with GSD is supersaturated with cholesterol (2, 9, 11–14). We hypothesized that such comparisons of the relative mol% data of GB lipids (12, 13, 15, 16) do not provide information as to whether cholesterol supersaturation results from excess hepatic secretion of biliary cholesterol, decreased biliary secretion of BAs or PLs, or a combination of both. We reasoned that straightforward mmol/l (millimole per liter) levels of GB bile lipids should instead provide more relevant direct information to answer this question.

To investigate this, we compared the mmol/l levels of GB lipids in GS and GS-free (GSF) patients with the results obtained using the corresponding mol% values. This was possible because we had access to data from a previously published report comprising 145 patients with GSD and 87 GSF patients, one of the largest patient materials published where pure GB bile, collected at surgery, was analyzed (12). In that article, the relative mol% for cholesterol, PLs, and BAs in GB bile was reported. We here expand that report with previously unpublished individual data on the mmol/l levels of GB lipids, also allowing for the evaluation of the correlations between GB bile CSI and the mmol/l levels of GB BAs, cholesterol, and PLs, respectively. Finally, we compared our results with those from 13 published studies where GB lipids were reported for GS and GSF patients from 11 countries, putting our results into a global perspective.

We show that in GSD globally, the mmol/l levels of BAs in GB bile are strongly reduced, while cholesterol is not increased. We conclude that the major cause for increased CSI in GSD is reduced BAs in GB bile.

## MATERIALS AND METHODS

### Collection of samples

GB bile was consecutively collected from the GBs of patients subjected to elective cholecystectomy between 1981 and 1998 as described in detail, including basal clinical data, in (12). The study was approved by the ethical committee at Karolinska Institutet and informed consent was obtained from all patients. There were 145 patients with cholesterol GSs (111 women, 34 men) and 87 GSF (73 women, 14 men). The GSF patients were cholecystectomized due to polyps, adenomyomatosis, or because of recurrent symptoms suggesting GB dysfunction. The BMI of the GSD patients was significantly higher (*P* < 0.05) than for controls (24.8 ± 0.3 kg/m^2^ vs. 23.5 ± 0.4 kg/m^2^). GB bile samples were analyzed for cholesterol, total BAs, PLs, and total lipids. Individual BAs were analyzed with gas-chromatography after alkaline hydrolysis as described (12). Cholesterol saturation of GB bile was calculated as described by Carey (4). GB lipids and mol% data of GB BAs, PLs, and cholesterol were determined as described (12).

Data were also retrieved from the tabular data and graphs of 13 references (17). All sorts used in the original publications were kept. In two reports (15, 18), BAs, cholesterol, and PLs in GBs were only available in mol% together with total lipid levels in milligrams per deciliter. In those studies, we calculated the corresponding levels in mmol/l assuming the molecular weights of BAs, cholesterol, and PLs as being 491, 387, and 775, respectively. All data presented are mean and SD. In one study referred to (19) (Fig. 2, panel D1), only mean data were available for GB mol% data. Differences between groups were determined by unpaired Student’s *t*-test using GraphPad Prism 7 version 7.03.

## RESULTS

**Fig. 1.**
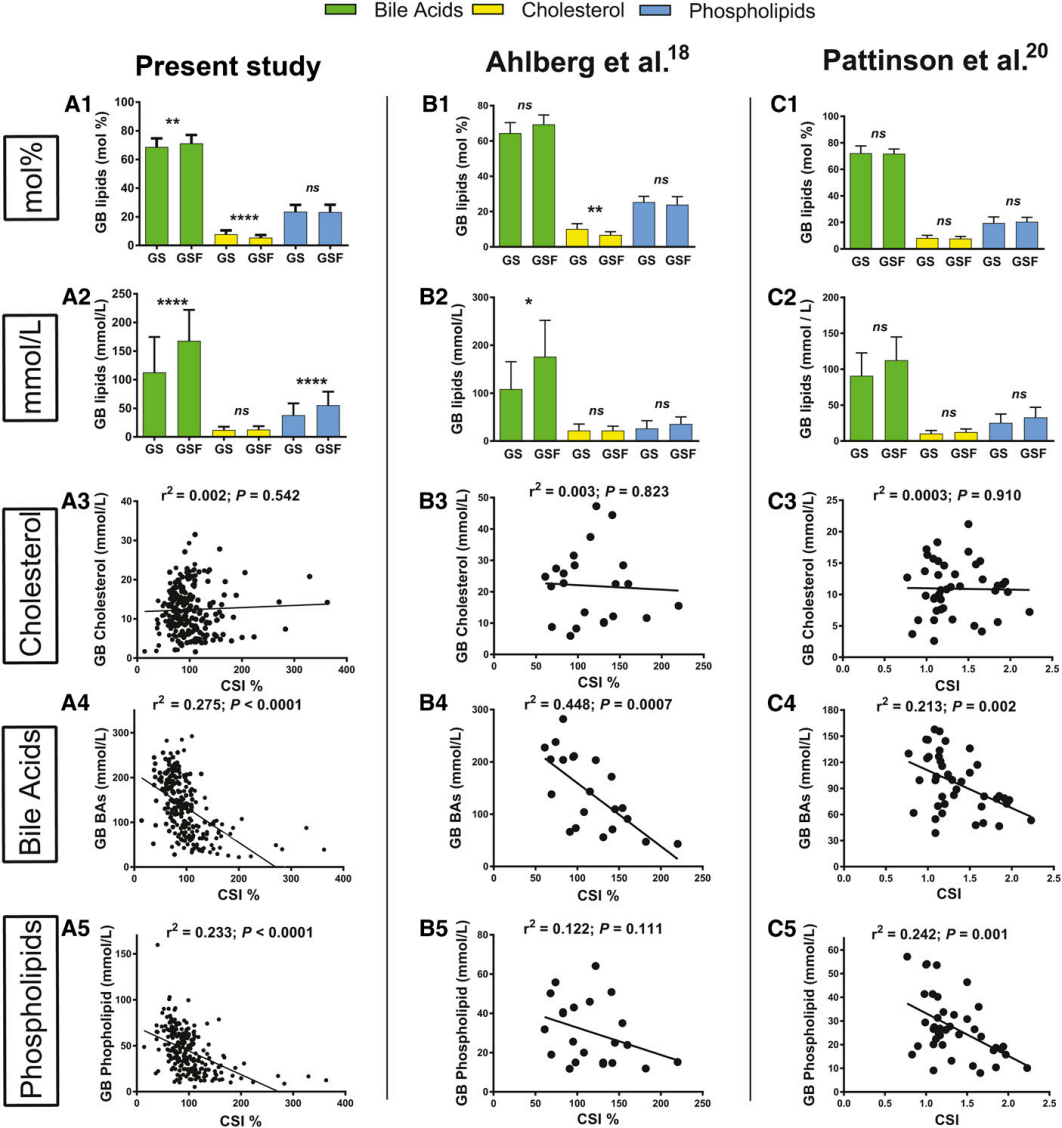
GB bile lipids in the present study and in two other studies (18, 20) where individual data were available. A1, B1, C1: Presented as mol%. A2, B2, C2: Shown as mmol/l. A3, B3, C3: Correlations between CSI and total GB bile cholesterol. A4, B4, C4: Correlations between CSI and total GB bile BAs. A5, B5, C5: Correlations between CSI and total PLs. Means and SD are shown with *P* values (*P < 0.05, **P < 0.01; ***P < 0.001; ****P < 0.0001; ns, not significant) from unpaired Student’s *t*-test between GS and GSF patients; r^2^ = Pearson’s correlation coefficient. Patient numbers are found in Table 1 and the Results.

We first compared GB lipids in GS and GSF patients shown in mol% (**Fig. 1**, panel A1) versus that in mmol/l (Fig. 1, panel A2). The mol% of BAs was slightly but significantly (*P* = 0.002) lower in GB bile from GS patients (68.7 ± 0.50%) compared with GSF patients (71.2 ± 0.63%), while the mol% of cholesterol was higher (7.8% vs. 5.5%, *P* < 0.0001) in GS patients, while PLs were unaltered. When the corresponding mmol/l data were examined, BAs and PLs were 33% and 31% lower, respectively (both *P* < 0.0001), in GB bile from GS patients, while GB cholesterol was unaltered in GS patients (*P* = 0.265). CSI% (CSI percent) was 52% higher (*P* < 0.0001) in GS patients (113 ± 3.85) compared with GSF patients (74.6 ± 2.55) (mean ± SEM). Because supersaturated bile is a prerequisite for the precipitation of cholesterol GSs and precedes GS formation (10), we next investigated the correlation between CSI of GB bile and GB mmol/l levels of cholesterol in the individual patients (Fig. 1, panel A3). There was no correlation (*P* = 0.542). In contrast, a correlation was found between the CSI of GB bile and the GB mmol/l level of BAs (*P* < 0.0001) (Fig. 1, panel A4). A strong correlation (*P* < 0.0001) was also present between GB CSI values and PL mmol/l levels (Fig. 1, panel A5).

### Comparisons of present results with those from 13 published reports

We next compared our findings with those from two studies on GB lipids in subjects with and without GSD where individual data on GB lipids were reported. First, in a Swedish study by Ahlberg, Angelin, and Einarsson (18), the individual mol% of GB BAs, cholesterol, and PLs was reported together with total GB lipids and CSI (Fig. 1, panels B). There were 10 GSF controls (8 women and 2 men) and 12 GS patients (9 women and 3 men). Because mmol/l data were not available, we calculated them inasmuch as total GB lipids were given. When data were expressed in mol%, the GB BAs’ relative values were on borderline significance (*P* = 0.056) for a slight reduction in GS patients (64.5% vs. 69.4% in GSF) (Fig. 1, panel B1). PLs were unchanged, while GB cholesterol relative values were increased in GSD (10.2% vs. 6.8%) (*P* = 0.005). When data were shown in mmol/l, BA levels were 38% lower in GB bile from GS subjects (*P* = 0.027), while cholesterol levels were unaltered (*P* = 0.968) (Fig. 1, panel B2). PL levels were 27% lower in GS patients but nonsignificant. The calculated CSI% was 52% higher (*P* = 0.004) in GS patients (138 ± 11.11) compared with GSF (91.1 ± 8.25) (mean ± SEM). We next evaluated the correlations between GB CSI and mmol/l levels of GB cholesterol and found, in line with the above results, no correlation (*P* = 0.823) (Fig. 1, panel B3). However, also in this study, there were strong correlations between CSI and GB mmol/l levels of BAs (*P* = 0.0007) (Fig. 1, panel B4), but not between CSI and GB mmol/l of PLs (*P* = 0.111) (Fig. 1, panel B5).

Second, in a study from New Zealand by Pattinson, Willis, and Frampton (20), individual GB BAs, cholesterol, and PLs in both mol% and mmol/l levels and CSI values were reported for 31 cholecystectomized GS patients and 10 GSF controls (Fig. 1, panels C). GB lipids in mol% showed no differences for any of the lipids (Fig. 1, panel C1). When expressed in mmol/l, BAs were 19% lower in GS patients, on borderline significance (*P* = 0.070), while cholesterol and PLs were 16% (*P* = 0.202) and 23% lower (*P* = 0.102), respectively, in GB bile from GS patients (Fig. 1, panel C2). In this study, CSI was not significantly different between GS (1.4 ± 0.06) and GSF (1.2 ± 0.10) (mean ± SEM) subjects, presumably due to the fact that 8 of the 10 control subjects had supersaturated bile. Importantly, there was no correlation between CSI and mmol/l levels of cholesterol (*P* = 0.910) (Fig. 1, panel C3). However, there was a clear correlation between CSI and the mmol/l levels of GB BAs (*P* = 0.002) and between CSI and mmol/l of PLs (*P* = 0.001) (Fig. 1, panels C4 and C5).

**Fig. 2.**
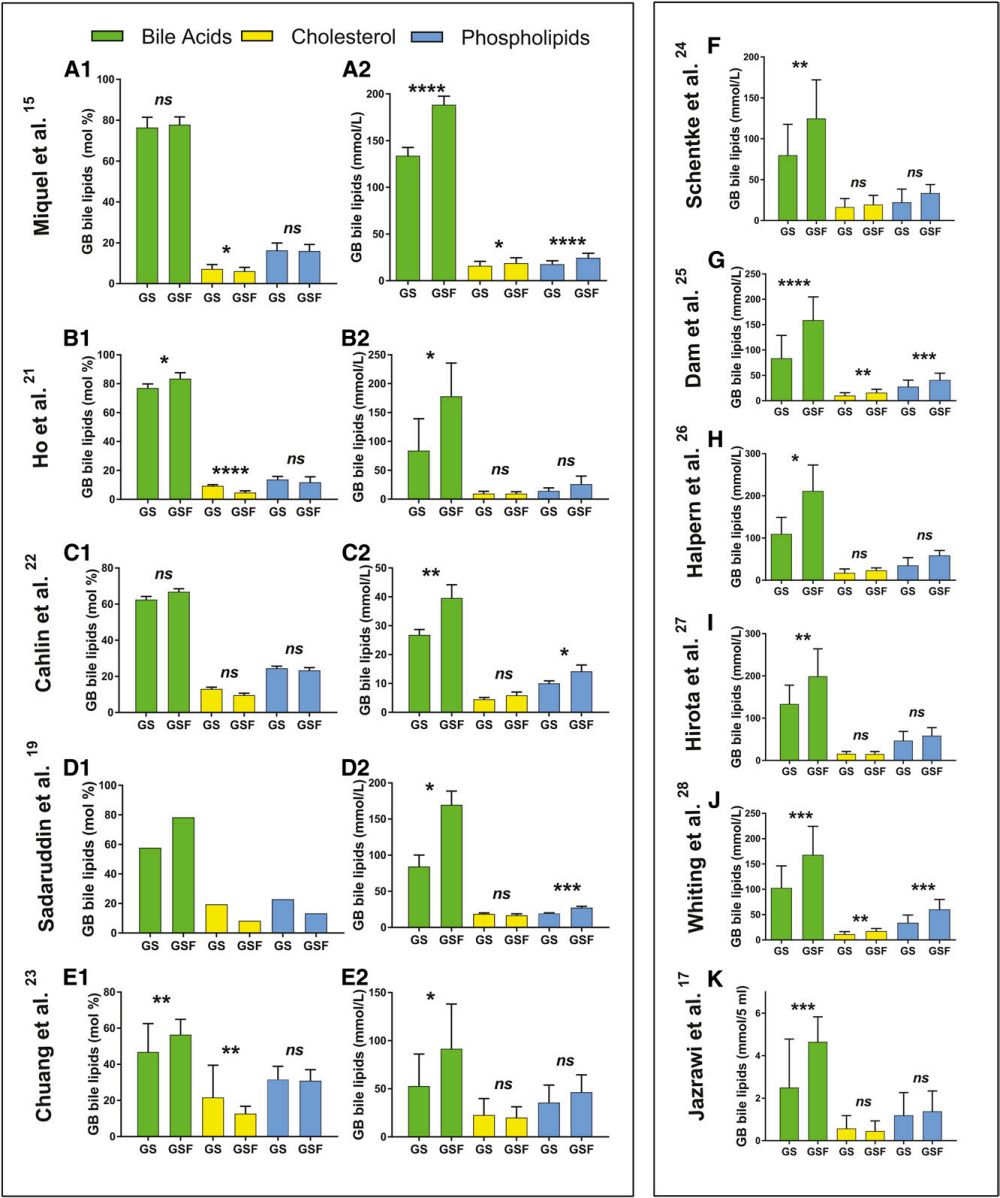
GB bile lipids in 11 studies. A–E: GB bile lipids from five studies on GS and GSF patients where mol% and mmol/l levels were reported. The results are presented row-wise and indicated by first author name and reference number. F–K: GB bile lipids from six studies on GS and GSF patients where only mmol/l levels were reported. The studies are indicated by first author name and reference. See Table 1 for the number of patients. The *P* values (*P < 0.05, **P < 0.01; ***P < 0.001; ****P < 0.0001; ns, not significant) from unpaired Student’s *t*-test between GS and GSF subjects are indicated.

We next analyzed five studies where mean mol% and mmol/l levels of GB lipids were available. First, in a study by Miquel et al. (15) on 52 Chilean GS patients and 40 GSF subjects, the mol% data were reported along with total GB lipids. When those data were presented in mol% (**Fig. 2**, panel A1), GB cholesterol values were 18% higher (*P* = 0.013) in patients with GSD (7.2 mol% vs 6.1 mol%), while BAs and PLs were unaltered as compared with GSF subjects. When we calculated the corresponding mmol/l levels from these data, GB BAs and PLs in subjects with GSD were then both significantly (*P* < 0.0001) reduced by 29% and 28%, respectively (Fig. 2, panel A2). Interestingly, GB mmol/l of cholesterol was now 15% lower (*P* = 0.012) in GB bile from GS patients compared with GSF patients (Fig. 2, panel A2), results similar to the three above studies. CSI% in the GS patients (130 ± 5) was 19% higher (*P* = 0.011) than in GSF subjects (109 ± 7) (mean ± SEM).

Second was a study by Ho et al. (21) from Taiwan (Fig. 2, panels B). The composition of GB bile from 10 controls and 4 patients with mixed stones was reported. When shown in mol% (Fig. 2, panel B1), GB BAs were lower (*P* = 0.014) in GS patients than in GSF individuals. PLs were unaltered, but the mol% of cholesterol was doubled (*P* < 0.0001). When results were shown in mmol/l (Fig. 2, panel B2), BAs were 53% lower (*P* = 0.015) in GB bile from GS patients and PLs were 45% lower in GB bile from GS patients but not significant. Cholesterol was unaltered. CSI% was 91% higher (*P* = 0.0005) in GS patients (201 ± 23) than in GSF subjects (105.1 ± 23) (mean ± SD).

Third, in a Swedish study by Cahlin et al. (22) (Fig. 2, panels C1 and C2), GB bile mol% data were evaluated. There were no differences for any of the three major GB lipids, but there was a 35% increase in cholesterol in GB bile on borderline significance (*P* = 0.061). However, when the mmol/l levels were examined, GB BAs were 32% lower (*P* = 0.005) in the GS group. PLs (lecithin) were 30% lower (*P* = 0.043), while cholesterol was 24% lower in the GS group, although not significant. There were no CSI data in this study.

Fourth, we retrieved a study by Sadaruddin, Hassan, and Zuberi (19) (Fig. 2, panels D1 and D2) on 23 Pakistani patients with mixed stones and six controls. Only GB lipid mean values without variations were reported for the mol% data. Nevertheless, the mean mmol/l data for GB lipids were in line with the above reports. Thus, the GB mmol/l of BAs were reduced by 50% (*P* = 0.036) in the GS group and PLs by 29% (*P* = 0.0007), while the mmol/l levels of GB cholesterol were unaltered. There were no CSI data in this study.

Fifth, was an American report by Chuang et al. (23) (Fig. 2, panels E1 and E2) where GB levels in mol% and mmol/l and CSI were reported from a study on 42 GSF subjects (14 men/28 women) and 11 GS patients (2 men/9 women). The mol% for BAs were 17% lower in the GS patients (*P* = 0.009), and cholesterol was 133% higher (*P* = 0.004), while PLs did not differ between GS and GSF subjects. However, when presented in mmol/l, the GB BAs in GS patients were 42% lower (*P* = 0.012), while cholesterol and PLs were unaltered. The CSI of GB bile was increased by 82% in GS patients (3 ± 3 vs. 1.7 ± 0.56) (mean ± SD) (*P* = 0.002).

We also analyzed six studies where only mmol/l concentrations of GB lipids were reported. The first was a German report by Schentke, Jaross, and Trubsbach (24) (Fig. 2F) on 18 GS and 10 GSF patients. The mmol/l levels of GB BAs were 36% lower (*P* < 0.010) in GS patients, while GB bile cholesterol did not differ between the two groups. PLs were 34% lower in the GS group but nonsignificant (*P* = 0.057). There were no CSI data. The second was a Danish study by Dam et al. (25) (Fig. 2G) on 26 GS and 27 GSF subjects. GB bile from GS patients had 47% lower mmol/l BA levels (*P* < 0.0001), 36% lower cholesterol levels (*P* = 0.003), and 32% lower PL levels (*P* = 0.001) than bile from the GSF subjects. There were no CSI data. The third study by Halpern et al. (26) (Fig. 2H) was from the USA on six GS patients and three GSF controls. Despite being a small study, the GS patients’ GB mmol/l levels of BAs were 48% lower (*P* = 0.018), and cholesterol and PLs were unaltered, although the latter showed a nonsignificant 41% decrease as compared with GSF patients. The CSI of GB bile was 41% higher but nonsignificant (*P* = 0.074) in the GS patients (1.5 ± 0.14 vs. 1.1 ± 0.02) (mean ± SEM). The fourth was a Japanese study by Hirota et al. (27) (Fig. 2I) on 16 GS patients and nine GSF controls. GB bile from the GS patients had 33% lower mmol/l BA levels (*P* = 0.007), while cholesterol and PLs were not altered. The CSI of GB bile was higher (*P* = 0.0007) in the GS patients (1.1 ± 0.26 vs. 0.7 ± 0.14) (mean ± SD). The fifth was an Australian study by Whiting and Watts (28) (Fig. 2J) where 18 GS patients and 14 GSF controls were studied. GB BAs, cholesterol, and PLs were all highly significantly reduced in the GS patients by 39, 34, and 43%, respectively. There were no CSI data. The sixth was an English study by Jazrawi et al. (17) (Fig. 2K) where 45 GS and 19 GSF patients were reported. As compared with GSF patients, GB bile from GS patients showed 46% reduced mmol/5 ml levels of BAs (*P* = 0.0003), while the levels of cholesterol and PLs were unaltered. CSI of GB bile from GS patients was 75% higher in GS patients (1.7 ± 0.1 vs. 0.97 ± 0.06) (mean ± SEM) (*P* < 0.0001).

Finally, for a robust overview of the results from the 13 studies referred to and our current data in Fig. 1A, we presented them all in **Table 1**.

**TABLE 1.** GB lipids in mol% bring focus on cholesterol in GSD, while cholesterol mmol/l levels in GB bile are never increased in patients with GSD

First Author (Reference)	Number	GB Lipids												CSI(percent increase in GS vs. GSF)	
		Percent Change of mol% Values in GS versus GSF Subjects						Percent Change of mmol/l Levels in GS versus GSF Subjects							
		Chol	*P*	BAs	*P*	PLs	*P*	Chol	*P*	BAs	*P*	PLs	*P*	Increase	*P*
Present study	232	**43%**	**<**0.0001	−*4%*	0.002	1%	0.751	−7%	0.265	−*33%*	**<**0.0001	−*31%*	**<**0.0001	**52%**	**<**0.0001
Ahlberg (18)	22	**50%**	0.005	−7%	0.056	6%	0.389	1%	0.968	−*38%*	0.027	−27%	0.175	**52%**	0.004
Pattinson (20)	41	6%	0.506	1%	0.833	−4%	0.589	−16%	0.202	−19%	0.070	−23%	0.102	16%	0.150
Miquel (15)	92	**18%**	0.013	−2%	0.147	2%	0.678	−*15%*	0.012	−*29%*	**<**0.0001	−*28%*	**<**0.0001	**19%**	0.011
Ho (21)	14	**98%**	**<**0.0001	−*8%*	0.014	16%	0.373	0%	**>**0.999	−*53%*	0.015	−45%	0.137	**91%**	0.0005
Cahlin (22)	27	35%	0.061	−6%	0.172	5%	0.551	−24%	0.241	−*32%*	0.005	−*30%*	0.043	No data	
Sadaruddin (19)	29	135%	Mean	−26%	Mean	72%	Mean	12%	0.514	−*50%*	0.036	−*29%*	0.0007	No data	
Chuang (23)	53	**133%**	0.004	−*17%*	0.009	2%	0.746	13%	0.540	−*42%*	0.012	−23%	0.081	**82%**	0.002
Schentke (24)	28	—	—	—	—	—	—	−15%	0.501	−*36%*	0.010	−34%	0.057	No data	
Dam (25)	53	—	—	—	—	—	—	−*36%*	0.003	−*47%*	**<**0.0001	−*32%*	0.001	No data	
Halpern (26)	9	—	—	—	—	—	—	−26%	0.365	−*48%*	0.018	−41%	0.088	41%	0.074
Hirota (27)	25	—	—	—	—	—	—	3%	0.840	−*33%*	0.007	−20%	0.187	**50%**	0.0007
Whiting (28)	32	—	—	—	—	—	—	−*34%*	0.003	−*39%*	0.0009	−*43%*	0.0002	No data	
Jazrawi (17)	64	—	—	—	—	—	—	27%	0.450	−*46%*	0.0003	−14%	0.507	**75%**	**<**0.0001
Total number of subjects	721														

Overview of differences in lipids in GB bile from patients with GSD compared with the respective data from GSF patients in the present study, and in 13 other reports indicated by first author and reference number. Left section, percent changes of mol% values for cholesterol, BAs, and PLs in GB bile from patients with GSD in relation to those for GSF patients. The rightmost section likewise shows the percent changes of the mmol/l levels of cholesterol, BAs, and PLs between GS and GSF patients. Boldface indicates significant increases; italic indicates significant reductions. *P* values are from unpaired Student’s *t*-test. In five studies, there were no CSI data. In one study (19), only means were reported. The percentage increases of CSI in GS patients versus GSF are indicated in the nine studies where CSI data were available. Chol, cholesterol.

## DISCUSSION

Much effort has been made to understand the prerequisites for cholesterol GS formation in the GB. Important landmarks are that GB bile must be supersaturated with cholesterol for GS precipitation (3), and that this precedes GS formation (3, 29–31). However, a high CSI value does not answer the question of whether high cholesterol and/or reduced BAs and/or PLs in the GB are causing it. We brought forward the mmol/l levels of GB bile lipids for a straightforward comparison to the respective mol% levels. To the best of our knowledge, such a comparison on a substantial number of patients has not been previously reported. We retrieved unpublished mmol/l data from a previous study (12), and compared our results with those from 13 published studies on GB lipids in GS and GSF patients. We made the following sets of important conclusions, all of global validity:

### GB BAs are strongly reduced in GSD

The mmol/l levels of GB BAs and PLs were reduced by 33% and 31%, respectively, in GS patients as compared with GSF subjects (Fig. 1, panel A2; Table 1). In line with these findings, 19–53% reduced GB mmol/l BA levels presented in patients with GSD in 12 of the 13 referred studies from 11 different countries, including Australia (28), Chile (15), China (21), Denmark (25), England (17), Germany (24), Japan (27), New Zealand (20), Pakistan (19), Sweden (12, 22), and USA (23, 26). GB mmol/l PLs were significantly reduced in five of the 13 referred studies (Table 1), while in the remaining eight studies, PLs were unaltered in GS patients.

### GB bile cholesterol levels are not elevated in GSD

GB cholesterol mmol/l levels in GS patients in the present study were not increased when compared with GSF patients (Table 1). In line with this, GB mmol/l cholesterol levels were not increased in any of the 13 studies referred to; in three studies, GB bile cholesterol was instead significantly reduced in GS patients as compared with GSF patients. These results, from measuring GB bile lipid mmol/l levels, are difficult to fit with the thinking that increased secretion of cholesterol into bile is the primary and major cause for why CSI is increased in GB bile in GSD. This is because, if cholesterol is secreted into bile at increased rates in GSD, this should presumably serve to increase the mmol/l levels of cholesterol in GB bile, which was not observed in any of the 14 studies.

### GB lipids mmol/l levels disclose a different picture than that from mol% data

When displayed in mol%, of the seven studies where mol% data and variations were available, mol% cholesterol was significantly increased in five (Table 1) and on borderline significance for an increase in one study [Table 1, Cahlin et al. (22)]. However, when shown in mmol/l, these six studies did not reveal any increases in GB bile cholesterol in GS patients; in one study GB cholesterol was instead reduced [Table 1, Miquel et al. (15); *P* = 0.012]. Further, in these six studies, mmol/l levels of BAs were significantly reduced by 29–53% (Table 1). Apparently, and in line with our hypothesis, comparing relative mol% data of biliary lipids (12, 13, 15, 16) results in severe misconceptions when attempting to identify the major, most common reason(s) for why GB bile is supersaturated with cholesterol in GSD.

### Supersaturated GB bile in patients with GSD is chiefly due to BA deficiency that precedes GS formation

The increased CSI of GB bile from GS patients was predominantly due to reduced BAs in GB bile (13 of 14 studies, Table 1) and to a certain extent to reduced PLs (6 of 14 studies), the latter in line with that the secretion of BAs modulates the secretion of PLs (32, 33). GB cholesterol levels apparently had a limited role in the increased CSI observed in the GS patients. Because it is well-established that GB bile must be supersaturated with cholesterol and that this presents prior to GS formation (3, 29–31), our results imply that reduced mmol/l levels of GB BAs and PLs also present prior to GS formation.

Overall, our findings in the present study and those from the 13 reports referred to consistently show that the major reason that CSI is increased in GS patients is because the actual mmol/l levels of GB BAs are reduced 19–53%. This is also in line with reports showing 30–50% reduced total BA pool sizes in GSD (29, 34–38). The lack of a major role of GB cholesterol mmol/l levels for elevated CSI in patients with GSD was further supported by the lack of correlation between CSI and mmol/l levels of GB bile cholesterol in the present study (Fig. 1, panel A3), as also seen in the reports by Ahlberg, Angelin, and Einarsson (18) (Fig. 1, panel B3) and by Pattinson, Willis, and Frampton (20) (Fig. 1, panel C3). Instead, there were strong negative correlations between CSI and BAs and PLs separately (Fig. 1, panels A4 and A5), confirmed in the reports by Ahlberg, Angelin, and Einarsson (18) and Pattinson, Willis, and Frampton (20). Indeed, all these reports (12, 15, 17–29, 34–37) show that GB BAs are deficient in GSD, evident when the actual mmol/l levels are considered, which does not align well with the view that the initial and major step in the generation of supersaturated bile is increased GB cholesterol due to elevated hepatic secretion of cholesterol fueled by hepatic overproduction of cholesterol (6, 8–10). The latter view is frequently linked to parallel comparisons of mol% data of GB bile lipids (12, 13, 15, 18). Surely, cholesterol secretion is increased in certain groups of patients with GSD. This is supported by reports on cholesterol secretion in GSD showing divergent results, from no differences in biliary lipid secretion (39) or reduced BA secretion with no changes in cholesterol secretion (40–42) to increased cholesterol secretion together with reduced secretion of BAs in GSD (43). When the latter report is cited for demonstrating increased cholesterol secretion in GSD, it seems as if it has been overlooked that this frequently cited study (104 citations) also contains data showing strong reductions in the secretion rates for BAs in GSD. Eight GS women were compared with 14 GSF. Cholesterol secretion was 43% higher in GS women (0.77 vs. 0.54 mg/h/kg). At the same time, BA secretion was reduced by 51% in GS patients (10.2 vs. 20.9 mg/h/kg). Thus, reports with cholesterol secretion data reveal results in line with our present findings of a major deficiency of BAs in GSD.

Why then are BAs deficient in GB bile in patients with GSD? This is an important question to answer in future studies. We can only speculate on this based on published reports. BA deficiency can develop from reduced BA production or from a higher intestinal loss of BAs. Both ways will themselves lead to a lower return of BAs from gut to liver at steady state. In the search for an anticipated reduced production of BAs in GSD, it was unexpectedly found that BA synthesis in 41 patients with GSD was 40% higher than in 72 GSF controls (44). Similar results have been published by Sauter and colleagues (45, 46); from investigation of 106 GSF (46) and 51 GS patients (45), BA synthesis was 31% higher in GS patients. Further, in a study on 165 Chilean women (47), BA synthesis was 37% higher in women with GSD as compared with GSF women. These four studies on 435 subjects from Sweden, Germany, and Chile consistently show that BA synthesis is significantly induced by 31–40% in patients with GSD. Thus, reduced BA synthesis is not likely to explain why BAs are deficient in GB bile from GS patients. The remaining possibility then seems to be that the major primary event should be an enhanced fecal loss of BAs, as has indeed been reported for GSD (48). This should lower the total BA pool (29, 34–38), leading to a compensatory induced BA synthesis consuming liver cholesterol, which will induce cholesterol synthesis, a finding reported for patients with GSD (47, 49). A particular situation when fecal loss of BAs is increased is BA diarrhea, a condition reported to be strongly linked to increased risk for GSD (50). Thus, considering the above-mentioned findings, GSD may be a disease of intestinal origin to a larger extent than previously anticipated. Possible causes for elevated fecal losses of BAs may be genetic and/or due to environmental factors. Interestingly, recent genetic studies have identified that loss-of-function mutations in the SLC10A2 gene, encoding for apical sodium-dependent BA transporter (ASBT), demonstrates that reduced BA transport by ASBT is linked to GSD (51, 52). Regarding environmental factors, one that is overlooked is the level of intake of food mass (2, 53). Apparently, while reduced food intake reduces both fecal loss of BAs (2, 53) and BA production (54), increased food intake increases the loss of fecal BAs (2, 53) as well as BA production (54). Because overeating is a basal prerequisite for increased BMI, a very strong risk factor for GSD (55), it is of particular interest to note that BMI has been identified as a causal factor for GSD in a Mendelian randomization study (56). Evidently, the reason(s) why BMI associates so strongly to GSD certainly warrants further study.

### Strengths and weaknesses of the study

Because bile was operatively obtained at cholecystectomy in the current study, as well as in the 13 studies referred to, this eliminated uncertainties as to whether GSs were present or not. Another strength of our study is the unusually large number of patients (n = 232). The GB bile mmol/l levels of cholesterol in GB bile from GSD patients were neither elevated in our study nor in any of the 13 studies referred to. It may be speculated that this could in part be due to a hampered ability to concentrate bile in GBs from GS patients. However, such a possibility does not explain why there were no correlations between CSI and GB bile cholesterol mmol/l levels in contrast to the strong correlations seen between CSI and BA mmol/l levels in the three studies where individual data were available (Fig. 1, panels A4, B4, and C4).
